# Changes in skin temperature and behaviors of preweaning Holstein calves in a hot environment monitored by a multimodal tail-attached device

**DOI:** 10.3168/jdsc.2023-0515

**Published:** 2024-03-02

**Authors:** Eri Furukawa, Tomomi Ozawa, Shogo Higaki, Tomoko Suda, Yosuke Sasaki, Kyotaro Murayama, Michiko Noguchi, Koji Yoshioka

**Affiliations:** 1 National Institute of Animal Health, National Agriculture and Food Research Organization, Tsukuba, Ibaraki 305-0856, Japan; 2 Department of Animal and Dairy Sciences, University of Wisconsin–Madison, Madison, WI 53706; 3 Department of Agriculture, School of Agriculture, Meiji University, Kawasaki 214-8571, Japan; 4 Dairy Technology Research Institute, National Federation of Dairy Co-operative Associations, Fukushima 969-0223, Japan; 5 Laboratory of Theriogenology, School of Veterinary Medicine, Azabu University, Sagamihara, Kanagawa 252-5201, Japan

## Abstract

This study aimed to determine the applicability of a tail-attached device in monitoring animal-based indicators (ABI) associated with changes in environmental conditions in calves through investigating the relationship between sensor-derived ABI and the temperature-humidity index (THI). Furthermore, to identify effective ABI indicative of heat stress status, sensor-derived ABI of calves under differing heat stress levels based on rectal temperature (RT) were compared. The tail-attached device, which is capable of measuring skin temperature (ST), activity intensity, and roll angle along the longitudinal axis of the tail at 3-min intervals, was attached to 99 preweaning female Holstein calves for an average of 4 wk (26.4 ± 6.8 d). After selecting data from mild to hot days (daily average THI of ≥55), physiological (daily maximum tail ST) and behavioral (daily average activity intensity, daily total lying time, and daily total body position change) ABI were computed, and their relationship with the daily average THI was determined using piecewise regression analysis. Additionally, during the hot season, RT of 20 randomly selected tested calves were measured thrice a week (every 2.4 ± 0.5 d), and a comparison was conducted between the ABI of calves with normal RT (<39.5°C) and those with high RT (≥39.5°C), utilizing data from days characterized by potential heat stress (daily average THI of ≥75). During the study, ambient temperature (°C) and relative humidity (%) were recorded every 10 min using an automatic digital data logger, from which the daily average THI was calculated. Piecewise regression analysis identified THI breakpoints of 73.6 for tail ST, 79.1 for average activity intensity, 72.3 for lying time, and 79.1 for position change. All the tested ABI tended to increase as THI increased, and this trend was pronounced in tail ST, activity intensity, and position change after the breakpoint. These 3 ABI were higher in calves with high RT compared with those with normal RT, whereas lying time shared similar values between the RT groups. Overall, these findings suggest that the tail-attached device can simultaneously monitor both physiological and behavioral ABI in calves, and among the ABI, tail ST, activity intensity, and position change are the effective ABI indicative of heat stress status.

## Highlights

• Animal-based indicators in calves were monitored by a tail-attached device.
• Tail skin temperature increased with increasing THI.
• Activity intensity and body position change increased with increasing THI.
• These upward trends were pronounced after the respective THI breakpoints.
• These ABI were higher in calves with high RT than in normal calves.

## Graphical Abstract

**Figure:**
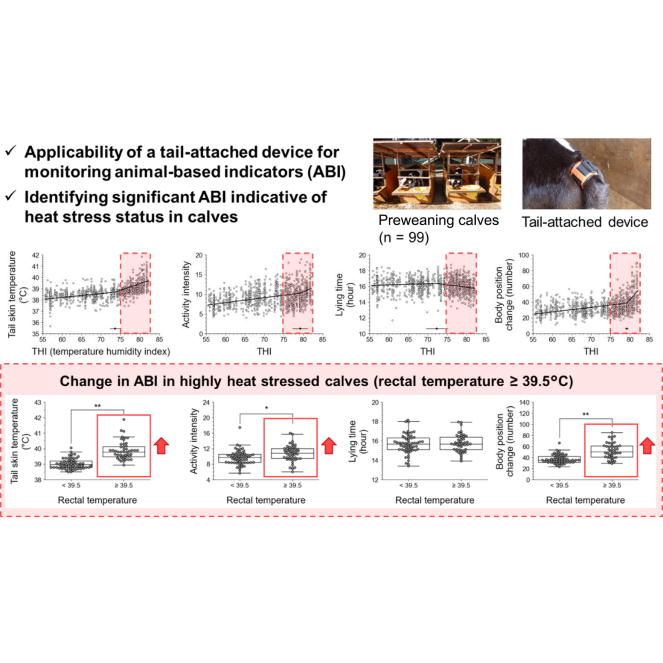
**Summary:** We determined the applicability of a tail-attached device in monitoring animal-based indicators (ABI) associated with heat stress in calves. Furthermore, we identified the ABI that best reflect heat stress status by comparing the sensor-derived ABI in calves with different rectal temperature (RT) levels. Daily maximum tail skin temperature (ST), daily average activity intensity, and daily total body position change showed pronounced increase after their respective temperature-humidity index (THI) breakpoints. Additionally, these 3 ABI were higher in calves with high RT than those with normal RT. These findings suggest that the tail-attached device can be used to monitor ABI in calves, and among the ABI, tail ST, activity intensity, and position change are the effective ABI indicative of heat stress status.

Global warming has emerged as an issue that can significantly affect dairy farm production (Berman, 2019). Although calves are not highly susceptible to heat stress due to their large surface-to-mass ratio and the absence of additional heat load related to lactation (Wang et al., 2020), heat stress can, nevertheless, negatively affect calf performance by reducing their feed intake (Dado-Senn et al., 2020) and decreasing their daily weight gain (Broucek et al., 2008). In general, heat stress is a significant concern for animal welfare (Polsky and von Keyserlingk, 2017). Therefore, the precise and timely detection of heat stress in calves is of critical importance for reducing economic losses and improving animal welfare.

Indicators of heat stress level in animals can be divided into 2 categories: environmental (e.g., the temperature-humidity index, **THI**) and animal-based (**ABI**), and between the 2, ABI are considered superior indicators because they reflect the actual heat stress status in individual animals (Islam et al., 2021). In cows, various physiological (e.g., respiration rate and body temperature) and behavioral (e.g., lying time and lying bout) parameters have been reported as possible ABI of heat stress (Hoffmann et al., 2020). However, information regarding ABI in calves is limited compared with cows. Most previous studies in calves have focused solely on physiological ABI such as respiration rate, heart rate, body temperature, and salivary cortisol (Dado-Senn et al., 2020, 2023; Kovács et al., 2020), or behavioral ABI such as lying time and bout (Kovács et al., 2018; Dado-Senn et al., 2022). Although studies such as Carter et al. (2014), Bakony et al. (2021), and Kamal et al. (2016) investigated both physiological (respiration rate and serum cortisol, and so on) and behavioral (proportion of standing and lying, and so on) ABI simultaneously, it remains unclear which ABI is the better indicator of heat stress level in calves. This limitation is primarily attributed to the lack of multimodal sensing devices applicable for calves (Costa et al., 2021).

Recently, a multimodal tail-attached device with a thermistor and 3-axis accelerometer was developed (Higaki et al., 2021) and validated for the simultaneous monitoring of physiological (tail skin temperature: tail **ST**) and behavioral (activity intensity, lying time, and body position changes; i.e., standing to lying or vice versa) parameters in cows (Higaki et al., 2021). However, this device's applicability in calves has not been established. Therefore, we aimed to determine the applicability of the tail-attached device for monitoring ABI associated with heat stress in calves by establishing the relationship between sensor-based ABI and THI. Furthermore, we identified the effective ABI indicative of heat stress status by comparing sensor-derived ABI of calves under differing heat stress levels based on rectal temperature (**RT**). Here, we tested the hypothesis that a tail-attached device is capable of monitoring ABI in calves, and we identified the effective ABI indicative of high heat stress levels in calves.

All procedures employed in this study were approved by the Institutional Animal Care and Use Committee of the National Institute of Animal Health, National Agriculture and Food Research Organization (protocol #21–032 and R4-R32-NIAH).

This experiment was conducted from August 2021 to July 2023 at a commercial dairy farm located in Japan, situated in a temperate humid climate zone. We enrolled a total of 99 healthy and preweaning female Holstein calves (16.8 ± 7.1 d old at the start of the experiment), which were individually housed in sawdust-bedded calf hutches measuring 1.8 m × 0.9 m without outdoor access. The calf hatch was constructed with wooden walls on all sides (front wall height: 0.5 m; side and rear walls: 0.9 m) and a plastic roof (height of 1.5 m in the front and 1.2 m in the rear). Ventilation was consistently facilitated through the space between the walls and the roof. These hutches were maintained under natural sunlight and temperature. Calves were fed twice daily with 6 L/d (at 12.3% solids) of commercial milk replacer (27% CP, 19% fat; Kurokke Super, Snow Brand Seed Co. Ltd., Sapporo, Japan) at 0600 and 1700 h. In addition, they were provided ad libitum access to a starter grain diet (18% CP; Low Carbo Starter, Snow Brand Seed Co. Ltd.) and chopped sudangrass hay in accordance with the recommendations of the Japanese Feeding Standard, as well as unrestricted access to water. During the experimental period, ambient temperature (°C) and relative humidity (%) were recorded every 10 min using an automatic digital data logger (TR-72wf, T & D Corp., Tokyo, Japan) placed in shade near the calf hutches. The THI was then calculated as follows: THI = 0.8 × T + [(RH/100) × (T − 14.4)] + 46.4, where T is dry bulb temperature and RH is relative humidity (Mader et al., 2006). The daily average THI was then calculated and this value was used for subsequent analysis.

We used the previously described multimodal tail-attached device equipped with a thermistor and 3-axis accelerometer (Higaki et al., 2021). The device, which measures 21.0 mm × 26.0 mm × 9.7 mm and weighs 5.8 g, including the battery, was attached as described previously (Higaki et al., 2021). In brief, the device was inserted into a pocket in a silicone rubber belt and sealed with a urethane gel sheet. The silicone rubber belt was wrapped around the tail base with the sensor positioned at the ventral side. The belt was then stabilized in place with a hook-and-loop fastener. The x-, y-, and z-axes of the 3-axis accelerometer were oriented to measure lateral, proximal/distal, and dorsoventral accelerations relative to the tail, respectively (Figure 1A). This device also measures ST (ranging from 20°C to 45°C), activity intensity (ranging from 0 to 102.3), and roll angle (i.e., rotation of the x- and z-axes about the y-axis, with values ranging from −3 to +3 rad; Figure 1B). The device wirelessly transmitted the data to the receiver in real time at 3-min intervals, which were then uploaded to the cloud server via 3G/LTE.

**Figure 1:**
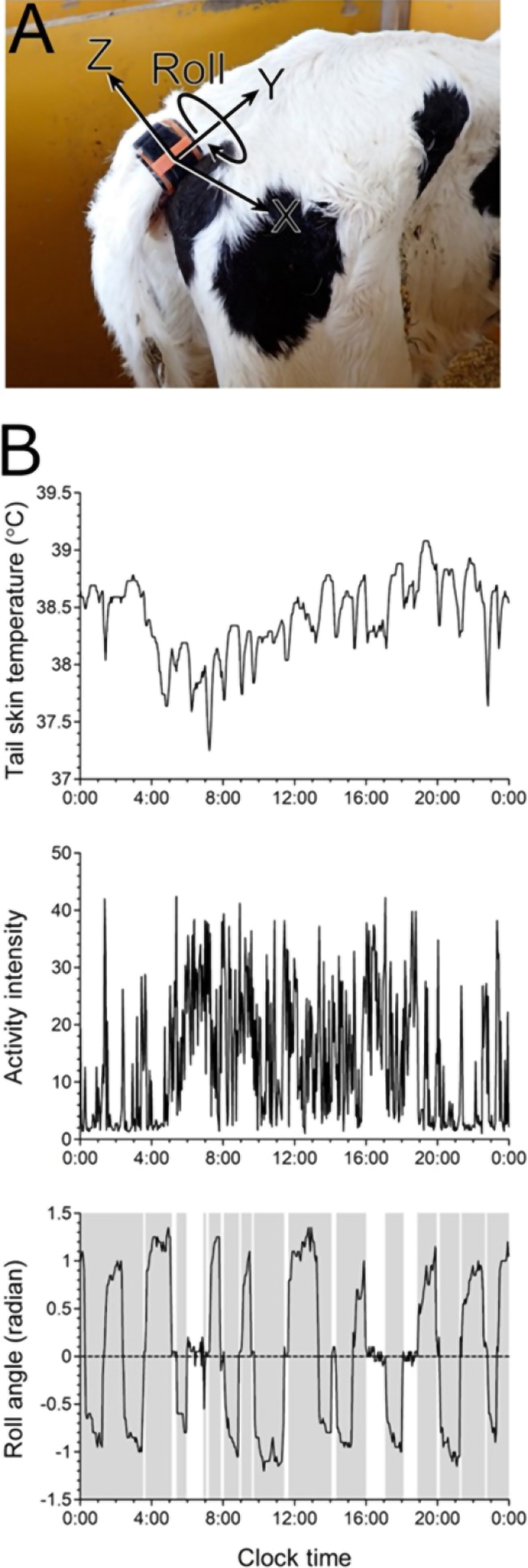
Tail-attached device used to generate sensor data and representative data collected over a 24-h period in a preweaning calf. (A) Photograph showing the position of the tail-attached device on a calf. The device is attached to the ventral side of the tail's base using dedicated equipment. It measures temperature and accelerations in the x-, y-, and z-axes, corresponding to lateral, proximal/distal, and dorsal/ventral accelerations relative to the tail, respectively. Roll indicates the rotation of the x- and z-axes about the y-axis. (B) Representative raw sensor data generated by the tail-attached device during a 24-h period in a preweaning calf. The top, middle, and bottom panels display tail skin temperature, activity intensity, and roll angle, respectively. Gray vertical bars indicate periods of time spent in a lying position.

The tail-attached device was attached to all calves, and sensor data were collected for an average period of 4 wk (26.4 ± 6.8 d); the calves reached an average age of 43.3 ± 10.1 d at the end of the experiment. Roll angle data were used to determine each animal's body positions (standing, lying left, and lying right) at each 3-min time point, as described in a previous study (Higaki et al., 2021) and using threshold values suitable for identifying the calves' body positions. Specifically, radians of ≥0.3, between −0.3 and 0.3, and <−0.3 corresponded to lying left, standing, and lying right body positions, respectively. We calculated the daily total lying time and total number of position changes (standing to lying down and vice versa and lying right to left and vice versa) based on the predicted position data. While the daily maximum ST was determined from the 3-min interval sensor data, the daily average activity intensity, daily total lying time, and daily total position changes were calculated from the 3-min interval data after excluding data at the time of feeding (0500–0659 and 1600–1759 h), due to its impact on the overall activity patterns of calves. During the potential heat stress period (summer to fall), we randomly selected 20 calves and measured their RT thrice a week (every 2.4 ± 0.5 d) at around 1100 h (between 1030 and 1130 h) using a digital thermometer (TV714J, Measure Technology Co. Ltd., Taipei, Taiwan).

In the analysis regarding the impact of hot environments on ABI, data from mild to hot days (daily average THI of ≥55; Hahn, 1999) were selected, and the resulting data from 99 calves were analyzed. The regression analysis of ABI on THI used the piecewise linear regression method (Muggeo, 2008), correcting for the fixed effect of age and the random effect of calf (Dado-Senn et al., 2023) as follows:

$$\begin{array}{l} ABI_{ij}=\beta_{0}+\beta_{1}THI_{ij}+\beta_{2}\left(THI_{ij}-THI_{bp}\right)THI_{k}\\ +\beta_{3}AGE_{ij}+CALF_{j}+\varepsilon_{ij}, \end{array}$$

$$[THI_{k}=\{\begin{array}{c} 0\;if\;THI\;\leq \;THI_{bp}\\ 1\;if\;THI\;>\;THI_{bp} \end{array}\;,$$

where *ABI_ij_* is the sensor-derived ABI, *β*_0_ is the population intercept, *β*_1_ is the left slope, *THI_ij_* is the daily average THI, *β*_2_ is the difference between the left and the right slopes, *THI_bp_* is the breakpoint, *THI_k_* is the dummy variable, *β*_3_ is the slope for age, *AGE_ij_* is the calf age (d), *CALF_j_* is an individual calf as a random intercept effect, and *ε_ij_* is the random error for the *i*th observation in the *j*th calf. An a priori sample size calculation was not conducted because there is no established calculation method for piecewise regression analysis.

The association between heat stress and sensor-derived ABI was evaluated by analyzing data obtained during days with a daily average THI of ≥75 (Hahn et al., 2009), consisting of 108 d of data from 20 individual animals. This data were then categorized into low and high heat stress groups, based on an RT threshold of 39.5°C (Bakony et al., 2023). This sample size was determined based on our preliminary data to detect a difference of 6.8 daily total position change (σ^2^ = 100) between the low and high heat stress groups when expecting the high RT (≥39.5°C) frequency of 21%. The estimated sample size was 103 d of data using 95% confidence and 80% power. The regression of ABI on RT used linear mixed models, which accounted for the fixed effect of age and the random effect of calf as follows:

$$ABI_{ij}=\beta_{0}+\beta_{1}RT_{ij}+\beta_{2}AGE_{ij}+CALF_{j}+\varepsilon_{ij},$$

where *RT_ij_* is the categorical RT levels of calves (<39.5°C or ≥39.5°C). Because the animal's core body temperature is maintained within a normal range until the heat load reaches a certain threshold level (Silanikove, 2000), the RT values of calves were divided into normal (<39.5°C) and high (≥39.5°C) temperature groups. No data in each RT group were excluded from the analysis. The model assumption of normality of residuals were visually examined using quantile-quantile plot. All statistical analysis was performed with R (version 4.3.0) using “nlme” and “segmented” packages (Muggeo, 2008). Differences were considered significant at *P*-values of <0.05. All values presented in the text represent the mean ± SD.

Attaching sensors to the calves did not result in any severe lesions throughout the experimental period, although all calves experienced temporary depilation. None of the calves exhibited severe symptoms of diseases requiring veterinary treatment.

The THI breakpoints for the daily maximum tail ST, daily average activity intensity, daily total lying time, and daily total position change were detected at 73.6, 79.1, 72.3, and 79.1, respectively (Figure 2). The ABI values at the corresponding THI breakpoints were as follows: the daily maximum tail ST was 38.7°C, the daily average activity intensity was 10.3, the daily total lying time was 16.4 h, and the daily total position change was 38.8 times, when adjusted for age to the average age of calves in the entire dataset, 27.7 d (Figure 2). The slope for the tail ST was considerably higher after the breakpoint (0.119) compared with before (0.035), suggesting a disruption in the regulation of body temperature under high THI conditions. We found no comparable data on the THI breakpoint for tail ST in the literature. However, Dado-Senn et al. (2020) reported the absence of THI breakpoints for rump, neck, and ear ST under a daily average THI ranging from approximately 60 to 90. Additionally, Dado-Senn et al. (2023) did not find a THI breakpoint for rump ST under a daily average THI ranging from approximately 61 to 78. In contrast, Kovács et al. (2020) reported a THI breakpoint of 83.0 for ear ST within a daily average THI range of approximately 76 to 92. The discrepancy regarding the presence of THI breakpoints and the relatively lower THI breakpoint for tail ST observed in this study indicate that the tail, particularly its ventral side, may have a limited capacity for heat dissipation compared with other exposed skin areas. This means that tail ST is a better indicator of core body temperature than the ST on other body parts, which is supported by the high correlation (R^2^ = 0.8) previously observed between the daily maximum tail ST and daily maximum RT (Nogami et al., 2013). Indeed, the THI breakpoint value that we observed for tail ST is similar to that for RT (69) determined under comparable THI conditions (i.e., daily average THI of 60.8–77.3: Dado-Senn et al., 2023). As RT is considered the standard for assessing homeothermy and heat stress (Dado-Senn et al., 2020), our results indicate that monitoring ST of the tail using a tail-attached device is a more reliable approach for assessing the heat stress levels compared with monitoring that of other body parts.

**Figure 2:**
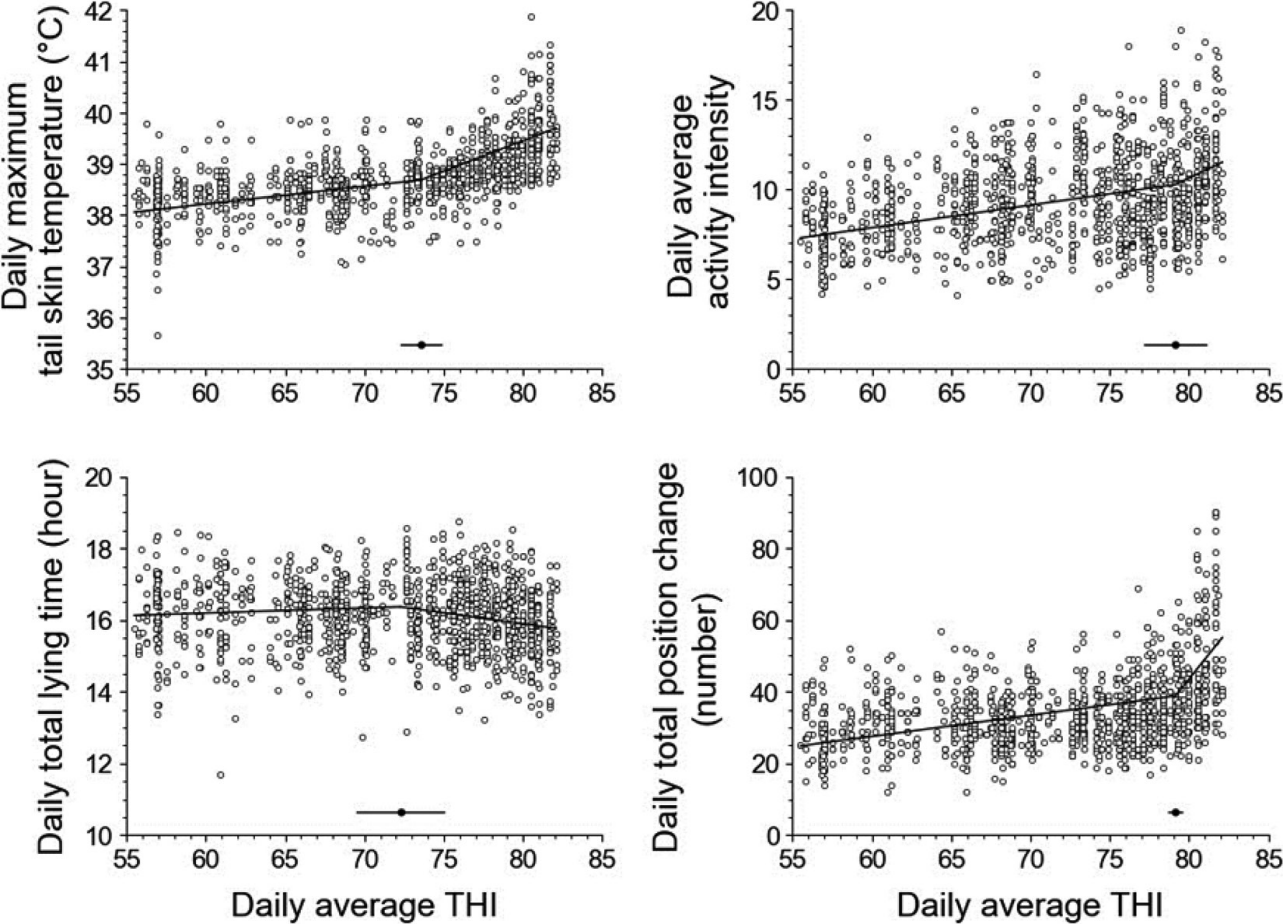
Relationship between daily average temperature-humidity index (THI) and sensor-derived animal-based indicators (ABI). Relationships between THI and the ABI were estimated using piecewise regression models. Open circles represent the observed values. Regression lines in each graph represent the predicted ABI derived from THI values in calves 27.7 d old (the average age of calves in the total dataset). Solid circles and lines above the x-axis represent breakpoints and their 95% CI, respectively.

Although we detected a breakpoint for daily total lying time, its slope remained nearly constant under the tested THI conditions (slopes before and after the breakpoint were 0.014 and −0.061, respectively). Meanwhile, the increases in daily total position changes during increasing THI tended to become more pronounced after the breakpoint (slopes before and after the breakpoint were 0.590 and 5.515, respectively). These findings align with those of previous studies. For instance, Kovács et al. (2018) reported that calves under nonshaded conditions (daily average THI of 78.1) changed their positions to lying down about 80% more frequently than calves under shaded conditions (daily average THI of 71.3), although the lying times of the nonshaded and shaded calves were similar. The pronounced increase in positional changes after the THI breakpoint likely reflects the increasing discomfort and frustration of calves under high heat stress (Kovács et al., 2018; Nordlund et al., 2019; Dado-Senn et al., 2022). This pronounced increase in position changes could explain the increase in daily average activity intensity observed above the THI breakpoint (slopes before and after the breakpoint were 0.126 and 0.419, respectively). However, Rice (2023) reported similar activity indices between days with high (≥70) and low (<70) daily average THI in calves monitored using a leg-attached accelerometer. This discrepancy may be attributable to the reference value of THI; namely, we set the breakpoint at a THI of 79, whereas Rice (2023) used a relatively low threshold (THI of 70).

The breakpoints for calves identified in the present study are consistent with the THI threshold of 75, as defined by the Associated Livestock Weather Safety Index (Hahn et al., 2009). Therefore, THI can serve as a valuable environmental indicator for effective heat stress management in calf herds. Interestingly, data from 108 d of monitoring 20 calves under high THI conditions (THI ≥75) included 61 d of data from 19 individuals that had normal RT (<39.5°C) and 47 d of data from 15 individuals that had high RT (≥39.5°C; Figure 3). Thus, based on heat stress conditions evaluated at a specific time of day considering the diurnal variation of RT, the present results indicated a lack of uniformity in the way that calves experience heat stress under high THI conditions, even in the same individual. However, more frequent RT measurements may be necessary in future studies to accurately determine the detailed heat stress conditions of individual calves. This is because the diurnal rhythm of RT could be affected by the microclimate of individual hutches (e.g., temperature and humidity; Dado-Senn et al., 2024), which was not assessed in this study. Nevertheless, the present results may indicate that the THI alone cannot be used to precisely detect heat-stressed individuals. In contrast, continuous monitoring of ABI is more useful for identifying heat-stressed individuals. In the present linear mixed models, the estimated coefficients of the RT group for the ABI were 0.60 (95% CI: 0.41, 078) for daily maximum tail ST, 0.78 (95% CI: 0.04, 1.52) for daily average activity intensity, 0.16 (95% CI: −0.17, 0.49) for daily total lying time, and 10.35 (95% CI: 5.87, 14.83) for daily total position change (Figure 3). Thus, we found that the daily maximum tail ST, daily average activity intensity, and daily total position change were higher in heat-stressed calves (RT ≥39.5°C) compared with normal calves (RT <39.5°C; *P* < 0.05), whereas the daily total lying time was similar in both groups. These findings suggest that among the ABI, tail ST, activity intensity, and position change are the reliable and effective ABI indicative of heat stress status in calves. Moreover, the tail-attached device can serve as a valuable tool for monitoring and detecting changes in these key ABI.

**Figure 3:**
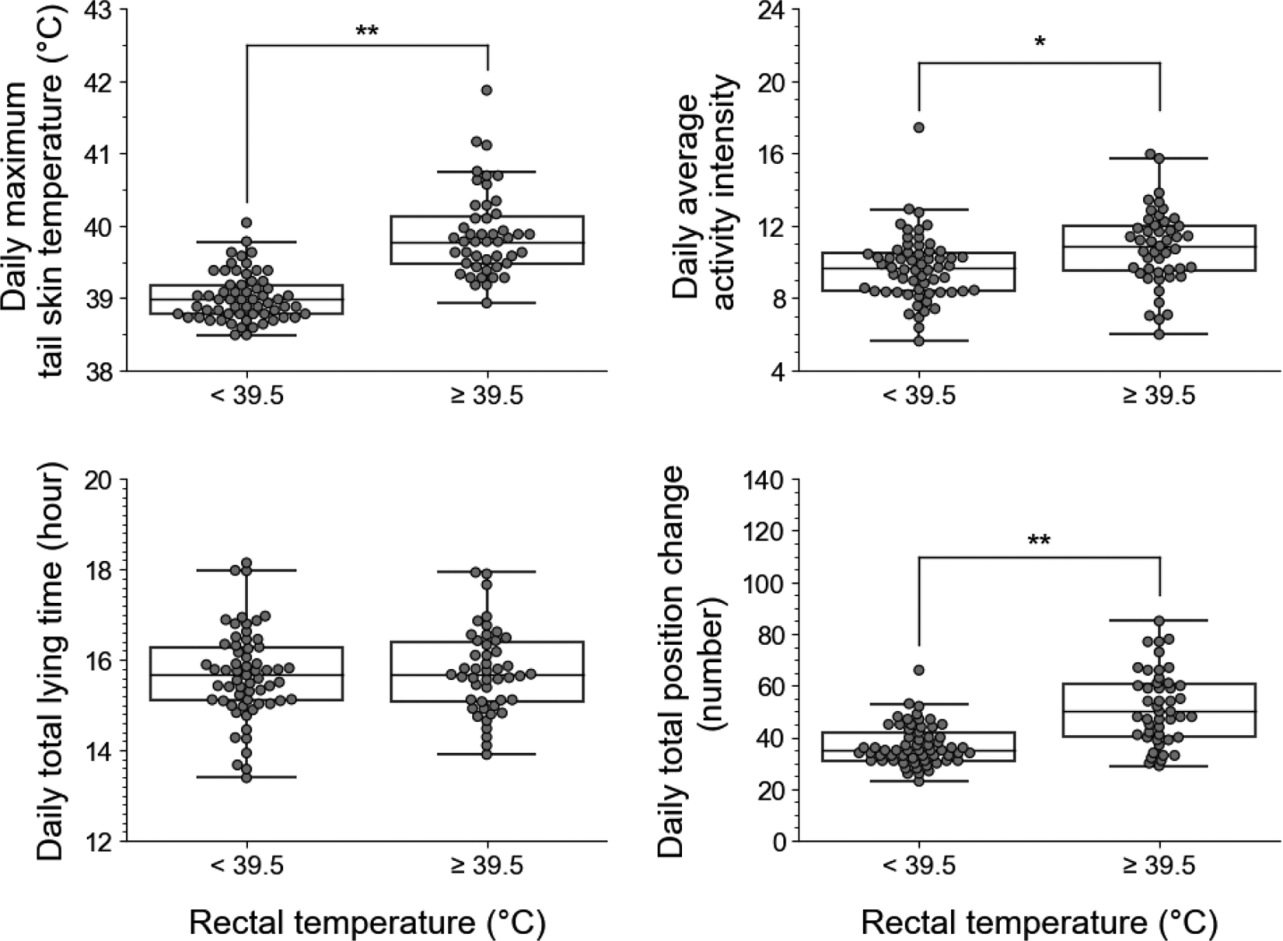
Relationship between rectal temperature (RT) levels and the sensor-derived animal-based indicators (ABI) in calves growing under high temperature-humidity index (THI) conditions. The relationship between heat stress levels and the sensor-based ABI was analyzed using calf data generated during days with a daily average THI of ≥75, with high and low heat stress groups defined by a threshold RT of 39.5°C. The relationship between RT levels (≥39.5°C and <39.5°C) and the sensor-based ABI was examined using linear mixed models. The results are displayed as a beeswarm boxplot, where the horizontal lines of the box indicate the quartiles and the whisker extends to 1.5 times the interquartile range or the maximum/minimum value, whichever is shorter. *P < 0.05; **P < 0.001.

In the present study, we examined the sensor-derived ABI for its association with THI as well as heat stress conditions, on a daily basis. This is because the ABI and THI over relatively short time periods varied and might not accurately reflect the actual heat stress status. Indeed, in mature cattle, it has been reported that the heat stress level is higher when both daytime and nighttime THI are elevated, compared with situations where only daytime THI is high (Mader et al., 2006). However, in actual farm situations, calves experiencing heat stress should be detected in a timely manner, to provide early intervention, lower morbidity and mortality rates directly and indirectly related to the heat stress, and thereby reduce the economic loss of the farm (Roland et al., 2016). Therefore, additional experiments should be conducted in smaller time intervals, through examining detailed sequential changes of ABI and comparing them with the heat stress status at specific intervals based on frequent RT measurements.

In conclusion, we have demonstrated the applicability of a tail-attached device for monitoring both physiological (tail ST) and behavioral (activity intensity, lying time, and body position change) ABI in preweaning calves. Among these ABI, tail ST, activity intensity, and position change are reliable and effective ABI indicative of heat stress status in calves. Use of the tail-attached device, with its ability to automatically and continuously monitor ABI in individual calves, has the potential to precisely detect heat-stressed calves in a timely manner, thereby reducing economic losses and enhancing animal welfare.
